# Natural killer cell-derived exosomal miR-1249-3p attenuates insulin resistance and inflammation in mouse models of type 2 diabetes

**DOI:** 10.1038/s41392-021-00805-y

**Published:** 2021-11-30

**Authors:** Ying Wang, Mengwei Li, Lin Chen, Huan Bian, Xiangying Chen, Huilin Zheng, Peiwei Yang, Quan Chen, Hanmei Xu

**Affiliations:** 1 The Engineering Research Center of Synthetic Peptide Drug Discovery and Evaluation of Jiangsu Province, China Pharmaceutical University, 210009 Nanjing, China; 2 State Key Laboratory of Natural Medicines, Ministry of Education, China Pharmaceutical University, 210009 Nanjing, China

**Keywords:** Molecular biology, Genetics

## Abstract

Natural killer (NK) cells have been suggested to be associated with type 2 diabetes by regulating systemic inflammation. However, the mechanism by which NK cells regulate insulin sensitivity remains unknown. This study shows that NK-derived exosomes from lean mice attenuate obesity-induced insulin resistance and inflammation in mice of type 2 diabetes. Moreover, lean NK-derived exosomes enhance insulin sensitivity and relieve inflammation in adipocytes and hepatocytes. MiR-1249-3p, which is significantly upregulated in lean NK-derived exosomes, can be transferred from NK cells to adipocytes and hepatocytes *via* exosomes. NK-derived exosomal miR-1249-3p dramatically induces cellular insulin sensitivity and relieves inflammation. Mechanistically, exosomal miR-1249-3p directly targets SKOR1 to regulate the formation of ternary complex SMAD6/MYD88/SMURF1, which mediates glucose homeostasis by suppressing the TLR4/NF-κB signaling pathway. This study reveals an emerging role for NK-derived exosomal miR-1249-3p in remission of insulin resistance, and provides a series of potential therapeutic targets in type 2 diabetes.

## Introduction

Type 2 diabetes (T2D), the most common form of metabolic diabetes mellitus,^1,2^ is characterized by high blood sugar (also called hyperglycemia) and insulin resistance. Individuals following a high-fat diet are particularly susceptible to obesity, which is clearly the most common cause of insulin resistance in humans.^3^ Symptoms of insulin resistance include the reduced sensitivity to insulin in adipocytes, muscle cells, and liver cells, as well as dysregulated insulin signaling and the metabolic disorders of glucose, fat, and protein, eventually causing a parallel rise of T2D.^4^ Patients with T2D are always companied by subclinical systemic low-grade inflammatory response^5^ and dysfunction of adipose tissue, liver, islet, hypothalamus, cardiac tissue, and other tissues, which ultimately develop into chronic complications.^6^

Previous studies have shown that immune system regulates metabolic organs throughout the body.^7^ Various immune cells such as T cells, B cells, macrophages, and NK cells, play vital roles in T2D.^8–12^ Obesity-induced inflammation, which is characterized by increased macrophage infiltration and associated with macrophage population shift from anti-inflammatory M2 to proinflammatory M1 macrophages, largely contributes to obesity-induced insulin resistance.^13^ Researchers have reported that NK cells and macrophages can cooperate to regulate inflammation.^14^ Moreover, adipocytes upregulate ligands of NK-cell-activating receptor (NCR1) in obesity, which triggers the proliferation of NK cells.^15^ Indeed, NK cells play a key role in obesity-induced inflammation and insulin resistance.^16,17^ However, the role and mechanism of NK cells in obesity-induced inflammation remain unclear.

Exosomes are vesicle-like bodies with a diameter of about 30–150 nm, that are multi-vesicles formed by the inward budding of the inner body membrane of cells, and released into the extracellular milieu by fusing with the cytomembrane.^18,19^ Exosomes are produced by various types of cells and serve as mediators in intercellular communication by transporting information cargos, such as proteins, lipids, and RNAs (miRNAs, mRNAs, lncRNAs, and circRNAs),^20–22^ which are stable in exosomes and transferred to adjacent and distal target cells.^23–25^ Moreover, exosomes can be secreted by islet beta cells, stem cells, and insulin-sensitive tissues, and then transferred to metabolic organs, immune cells, and endothelial cells to maintain glucose homeostasis or aggravate insulin resistance through immune response, oxidative stress, and angiogenesis.^26–28^ However, the involvement of splenic NK cell-derived exosomes in the occurrence and development of T2D remains unclear.

Our previous observation indicates that NK cells exert antidepressant-like effects in mice by controlling the release of inflammatory factors.^29^ NK cell-derived exosomal miR-207 decreases the release of pro-inflammatory cytokines (IL-1β, IL-6, and TNF-α) by astrocytes and alleviates depression-like symptoms in mice.^30^ In this study, we are interested in understanding the role of NK cells in metabolic regulation, insulin resistance, and inflammation occurring in T2D. Our studies reveal an emerging role for NK-derived exosomes in remission of insulin resistance, and provide potential therapeutic targets in T2D.

## Results

### NK-derived exosomes from lean mice attenuate obesity-induced insulin resistance

**Fig. 1:**
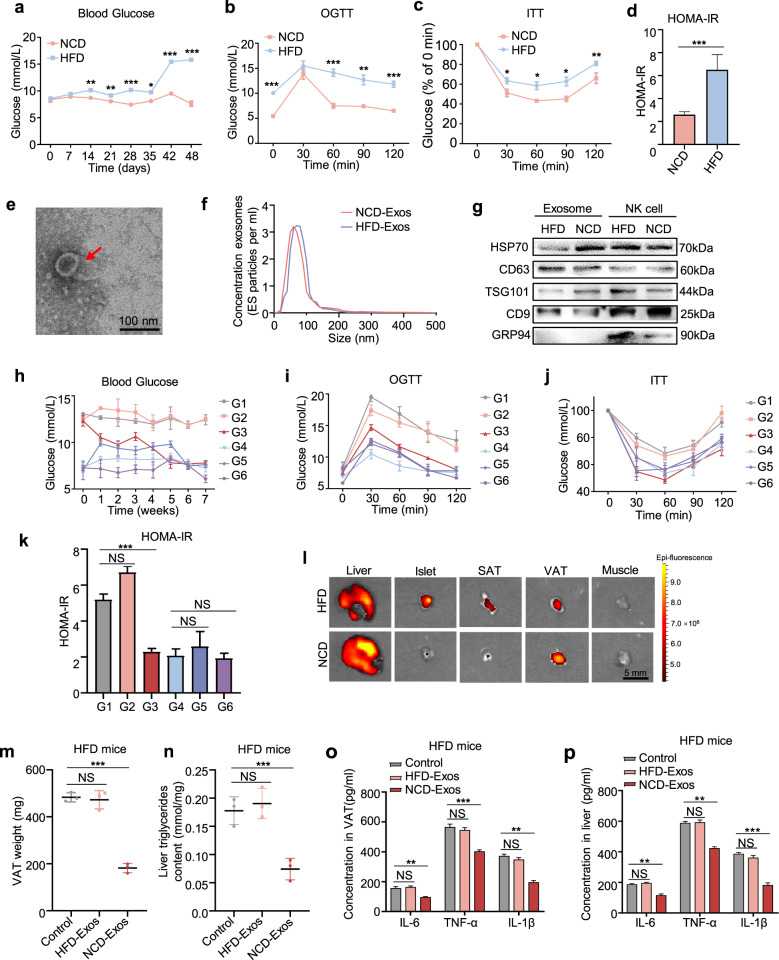
NK-derived exosomes from lean mice attenuate obesity-induced insulin resistance. **a** Fasting blood glucose of NCD and HFD mice after group feeding. **b**–**d** OGTT (**b**), ITT (**c**), and HOMA-IR index (**d**) of HFD mice and NCD mice (*n* = 48) were recorded at 42 days after group feeding. HOMA-IR = Fasting blood glucose value × fasting serum insulin value/22.5. **e** Transmission electron micrographs of NK-derived exosomes. Scale bar, 100 nm. **f** NanoSight particle tracking analysis showing the particle size of NK-derived exosomes isolated from NCD and HFD mice. **g** Western blot assays of exosomal markers TSG101, HSP70, CD63, and CD9. **h**–**k** After blank liposomes, NCD-Exos, and HFD-Exos were separately injected into NCD or HFD mice *via* tail vein, fasting blood glucose (**h**), OGTT (**i**), ITT (**j**), and HOMA-IR (**k**) were assessed in each group. G1: HFD mice treated with blank liposomes (*n* = 3); G2: HFD mice treated with HFD-Exos (*n* = 3); G3: HFD mice treated with NCD-Exos (*n* = 3); G4: NCD mice treated with blank liposomes (*n* = 3); G5: NCD mice treated with HFD-Exos (*n* = 3); and G6: NCD mice treated with NCD-Exos (*n* = 3). **l** After NCD-Exos labeled with PKH26 were transferred into recipient mice, fluorescence images of the liver, islets, SATs, VATs, and skeletal muscles were observed by in vitro imaging system. Scale bar, 5 mm. **m** The weight of VATs from HFD mice treated with NCD-Exos, HFD-Exos, or blank liposomes. **n** Liver triglyceride content of HFD mice treated with NCD-Exos, HFD-Exos, or blank liposomes. **o**, **p** ELISA assays of IL-6, IL-1β, and TNF-α expression in VATs and livers of HFD mice treated with NCD-Exos, HFD-Exos, or blank liposomes. Experiments were performed at least in triplicate, and the results are shown as the mean ± s.d. Student’s t-test was used to analyze the data. (*P < 0.05; **P < 0.01; ***P < 0.001)

To investigate the role of NK-derived exosomes in insulin resistance, a T2D mouse model was established using a high-fat diet (HFD) combined with low doses of streptozotocin (STZ), and mice in the control group were fed with normal chow diet (NCD) (Supplementary Fig. S1a). The body weight of HFD mice significantly increased (Supplementary Fig. S1b). As shown in the results of fasting blood glucose (Fig. 1a), OGTT (Fig. 1b), ITT (Fig. 1c), and HOMA-IR indices (Fig. 1d), HFD mice were insulin resistant. The AUC of the OGTT curve (Supplementary Fig. S1c) and fasting insulin levels (Supplementary Fig. S1d) further illustrated that we had successfully established a T2D mouse model.

NK cells were isolated from splenic lymphocytes by flow cytometry sorting (Supplementary Fig. S1e). Exosomes were isolated and purified from conditioned media of NK cells by ultracentrifugation. Electron microscopy (Fig. 1e) and NanoSight analysis (Fig. 1f) revealed that particles isolated from conditioned media of NK cells contain abundant exosomes with a diameter of 30–150 nm. In addition, the detection of exosomal markers HSP70, CD63, TSG101, CD9, and the absence of endoplasmic reticulum protein GRP94 further verified that the isolated particles were mainly exosomes (Fig. 1g), indicating that NK cells can secrete exosomes.

To identify the biological function of NK-derived exosomes, NCD mice and HFD mice were respectively treated with NK-derived exosomes from NCD mice (NCD-Exos) or NK-derived exosomes from HFD mice (HFD-Exos) *via* the tail vein. The body weight of HFD mice was reduced to a normal level by NCD-Exos (Supplementary Fig. S1f). Systemic insulin sensitivity was significantly increased in HFD mice treated with NCD-Exos as evidenced by the results of fasting blood glucose, OGTT, ITT, and HOMA-IR (Fig. 1h–k and Supplementary Fig. S1g–j). However, there was no significant effect in other groups. Therefore, we mainly focused on differences in effects between NCD-Exos and HFD-Exos in HFD mice. To observe the in vivo distribution of NK-derived exosomes, mice were treated with NCD-Exos stained with red fluorescent PKH26 through the tail vein. The results showed that NK-derived exosomes were mainly concentrated in visceral adipose tissues (VATs), subcutaneous adipose tissues (SATs), islets and liver, but much lower in muscle tissues (Fig. 1l). Moreover, the weight of the VATs and the level of liver triglycerides increased in HFD mice (Supplementary Fig. S1k, l), but were significantly relieved by NCD-Exos (Fig. 1m, n). In addition, the expression of pro-inflammatory factor genes was significantly downregulated by NCD-Exos in the VATs and livers of HFD mice (Supplementary Fig. S1m, n and Fig. 1o, p). However, there was no change in pro-inflammatory factor expression in the SATs and islets of HFD mice. Consistent with above results, insulin signaling was activated by NCD-Exos in the VATs and livers of HFD mice, as evidenced by increased phosphorylation of Akt (which can induce glucose transportation), PPARγ (which can increase insulin sensitivity), and GLUT4 (which can promote glucose transportation) (Supplementary Fig. S2a–d). Collectively, these results suggest that NK-derived exosomes from NCD mice attenuate obesity-induced insulin resistance and inflammation in VATs and livers.

### Lean NK-derived exosomes enhance cellular insulin sensitivity

**Fig. 2:**
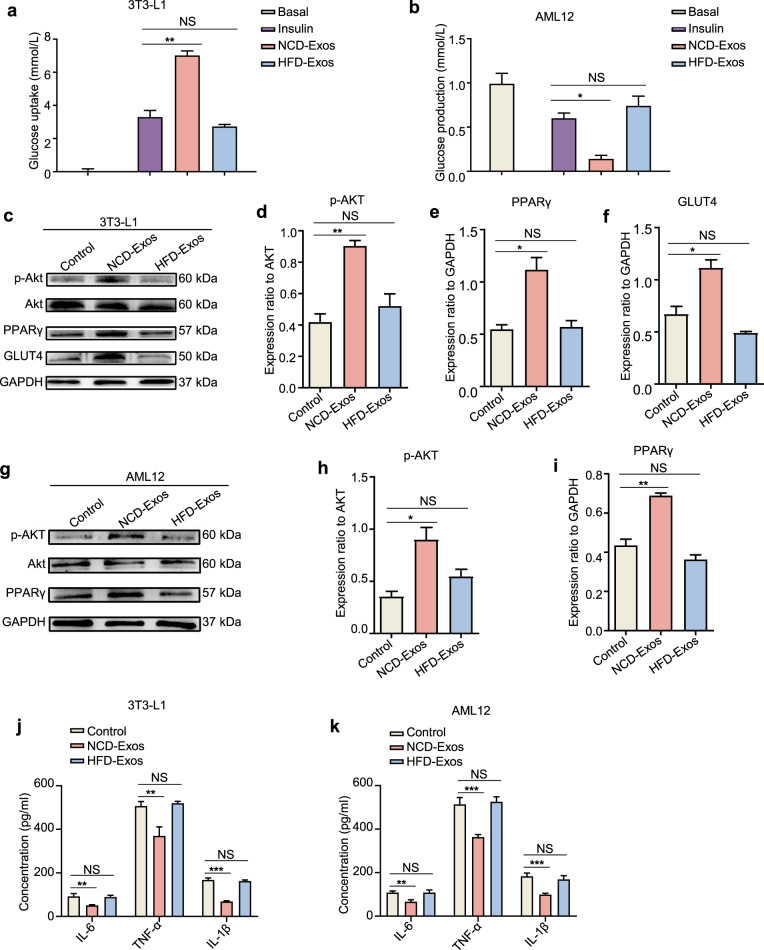
Lean NK-derived exosomes enhance cellular insulin sensitivity. **a**, **b** Two microgram NCD-Exos or HFD-Exos was added to the culture medium of 3T3-L1 adipocytes or AML12 cells. After 24 h of culture, glucose uptake content of 3T3-L1 adipocytes (**a**) and glucose output content of AML12 cells were measured (**b**). **c**–**f** Western blot assays of p-Akt, Akt, PPARγ, and GLUT4 expression in 3T3-L1 adipocytes treated with NCD-Exos or HFD-Exos. **g**–**i** Western blot analysis of p-Akt, Akt, and PPARγ expression in AML12 cells treated with NCD-Exos or HFD-Exos. **j**, **k** IL-1β, IL-6, and TNF-α expression in 3T3-L1 adipocytes (**j**) and AML12 cells (**k**) treated with NCD-Exos or HFD-Exos was assessed using ELISA. Experiments were performed at least in triplicate, and the results are shown as the mean ± s.d. Student’s *t*-test was used to analyze the data. (*P < 0.05; **P < 0.01; ***P < 0.001)

Given the prominent in vivo effects and distribution of NCD-Exos, we next investigated the in vitro function of NCD-Exos in adipocytes and hepatocytes. As shown in Fig. 2a, b, NCD-Exos elevated insulin-stimulated glucose uptake in 3T3-L1 adipocytes, and reduced glucose output in AML12 cells. In addition, the expression of p-Akt and PPARγ increased in 3T3-L1 adipocytes and AML12 cells with NCD-Exos, along with a consistent upregulation of GLUT4 expression in 3T3-L1 adipocytes (Fig. 2c–i). However, HFD-Exos had no obvious effect.

Moreover, protein secretion of pro-inflammatory factors such as IL-6, IL-1β, and TNF-α in 3T3-L1 adipocytes and AML12 cells was significantly downregulated by NCD-Exos (Fig. 2j, k). Overall, the in vitro results show that NCD-Exos improve insulin sensitivity and relieve inflammation in adipocytes and hepatocytes, which coincide with our in vivo results.

### NK-derived exosomal miR-1249-3p mediates cellular insulin sensitivity and inflammation

**Fig. 3:**
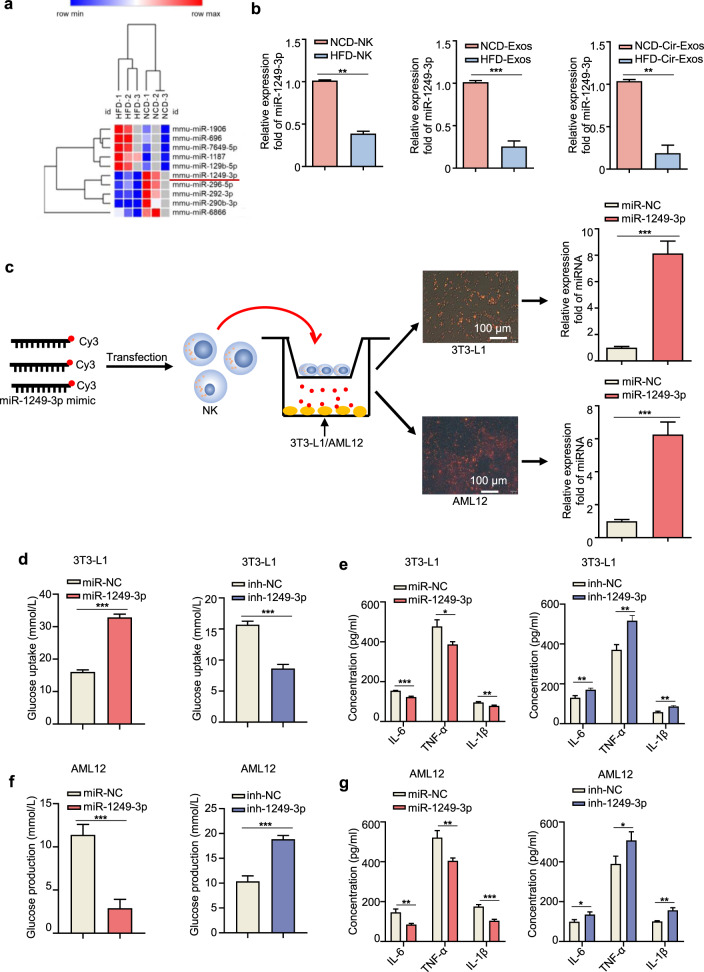
NK-derived exosomal miR-1249-3p mediates cellular insulin sensitivity and inflammation. **a** Microarray analysis of significantly expressed exosomal miRNAs between NCD-Exos and HFD-Exos was presented in a heatmap. **b** qRT-PCR assay of miR-1249-3p expression in splenic NK cells, NK-derived exosomes, and circulating exosomes from NCD or HFD mice. **c** NK cells transfected with a Cy3-labeled miR-1249-3p mimic were co-cultured with 3T3-L1 adipocytes or AML12 cells in a Transwell^TM^ plate (membrane pore = 0.4 mm) plate. Scale bar, 100 μm. **d**–**g** After transfecting with miR-1249-3p mimic, miR-NC, inhibitor, and inh-NC, the glucose uptake content of 3T3-L1 adipocytes (**d**) and glucose production content of AML12 cells (**f**) were measured, and the concentrations of IL-6, TNF-α, and IL-1β secreted by 3T3-L1 adipocytes (**e**) and AML12 cells (**g**) were detected by ELISA. Experiments were performed at least in triplicate, and the results are shown as the mean ± s.d. Student’s *t*-test was used to analyze the data. (*P < 0.05; **P < 0.01; ***P < 0.001)

We further explored the mechanisms by which NCD-Exos promote insulin sensitivity. Emerging evidence show that abundant miRNAs are encapsulated in exosomes and play an important role in cell–cell communications.^31^ Therefore, we hypothesized that NK-derived exosomal miRNAs participate in regulating insulin sensitivity in adipocytes and hepatocytes. To identify the specific miRNAs involved, we conducted microarrays to generate miRNA profiles of NCD-Exos and HFD-Exos. Comparison of differentially expressed miRNAs was calculated by single channel chip and normalized by Lowess, and the results were compared and shown as heatmaps in Fig. 3a. Supplementary Table S1 and Supplementary Fig. S3a show ten miRNAs with the most significant (fold change >1.5, *P* < 0.05) abundance difference between NCD-Exos and HFD-Exos, among which miR-1249-3p was most remarkably increased in NCD-Exos. In addition, targets of miR-1249-3p were directly involved in the metabolic pathway of Gene Ontology (GO) and Kyoto Encyclopedia of Genes and Genomes (KEGG) database (Supplementary Fig. S3b–e). Moreover, miR-1249-3p was upregulated in NK cells, NK-derived exosomes, and circulating exosomes from NCD mice relative to HFD mice (Fig. 3b). In addition, miR-1249-3p expression levels significantly increased in the VATs and livers of NCD mice (Supplementary Fig. S3f, g). Reasonably, we surmised that the differential expression of miR-1249-3p in VATs and livers was attributable to NK-derived exosome tropism.

Next, we verified whether exosomal miRNAs are secreted by NK cells and transported into target cells. After transfection of a fluorescent Cy3-labeled miR-1249-3p mimic, NK cells were co-cultured with 3T3-L1 adipocytes or AML12 cells in a Transwell^TM^ plate (Fig. 3c). The appearance of Cy3 dye in the 3T3-L1 adipocytes and AML12 cells demonstrated that the Cy3-miR-1249-3p mimic was delivered from NK cells in the upper Transwell^TM^ to the recipient adipocytes and hepatocytes seeded in the lower well. The Cy3 dye appearance was concomitant with an eight-fold increase in miR-1249-3p abundance in 3T3-L1 adipocytes after co-culture and six-fold increase in AML12 cells (Fig. 3c). In control experiments, we treated NK cells with Cy3 dye alone (not conjugated to miR-1249-3p) and did not detect the Cy3 dye in 3T3-L1 adipocytes and AML12 cells after co-culture (Supplementary Fig. S3h). Moreover, miR-1249-3p expression was significantly higher in circulating exosomes of NCD mice than that of NFD mice, which was possibly caused by exosome migration from splenic NK cells to target cells *via* the circulatory system (Fig. 3b). Overall, exosomal miR-1249-3p can be secreted by NK cells and taken up by adipocytes and hepatocytes.

In addition, we determined the effects of miR-1249-3p on insulin function. MiR-1249-3p mimic, inhibitor, and negative control were transfected into 3T3-L1 adipocytes and AML12 cells separately (Supplementary Fig. S3i, j). The results showed that overexpression of miR-1249-3p significantly increased insulin-stimulated glucose uptake in 3T3-L1 adipocytes (Fig. 3d), which was accompanied by downregulated expression of pro-inflammatory factors (Fig. 3e). In AML12 cells, transfection of miR-1249-3p mimic promoted the suppressive effect of insulin on glucose production (Fig. 3f), along with decreased pro-inflammatory factor expression (Fig. 3g). Taken together, the above results suggest that NK-derived exosomal miR-1249-3p from NCD mice promotes cellular insulin sensitivity and relieve inflammation.

### Exosomal miR-1249-3p directly targets SKOR1 to mediate insulin sensitivity

**Fig. 4:**
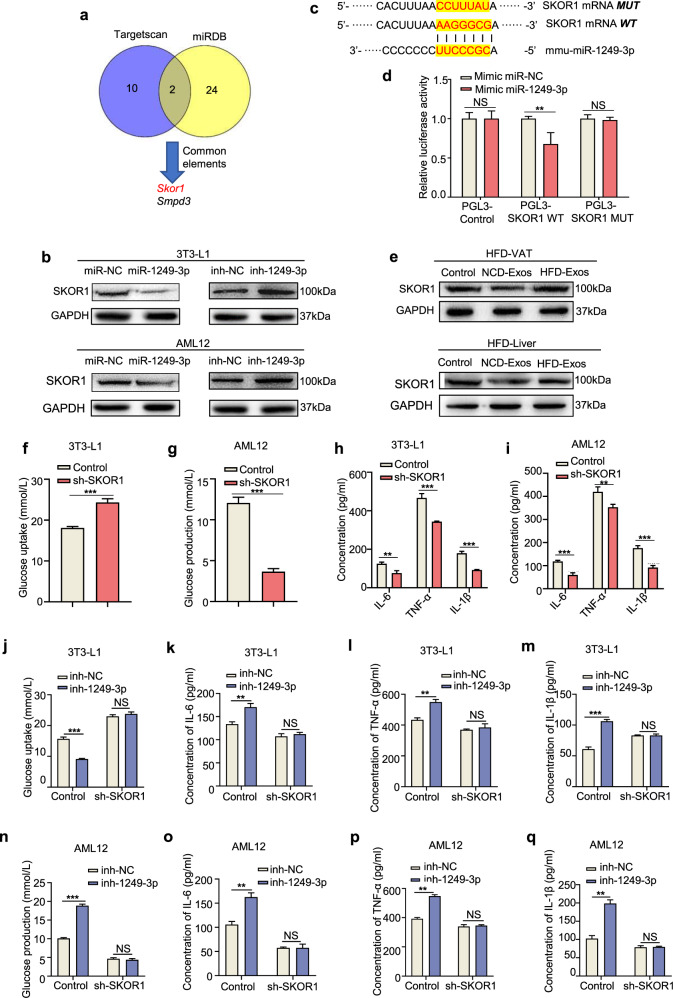
Exosomal miR-1249-3p directly targets SKOR1 to mediate insulin sensitivity. **a** The potential targets of miR-1249-3p were predicted by integrating the results of two databases (TargetScan and miRDB). **b** Western blot analysis of SKOR1 in 3T3-L1 adipocytes and AML12 cells with the indicated treatments. **c** The wild-type and a mutated type of binding site between miR-1249-3p and SKOR1. **d** Relative luciferase activity of AML12 cells in the presence of indicated treatments. **e** Western blot analysis of SKOR1 expression in the VATs and livers of HFD mice after NCD-Exos or HFD-Exos treatment. **f**–**i** The effect of sh-SKOR1 on glucose uptake capacity in 3T3-L1 adipocytes (**f**), glucose production capacity in AML12 cells (**g**), and expression levels of IL-6, TNF-α, and IL-1β (**h**, **i**). **j**–**q** Glucose uptake content of 3T3-L1 adipocytes (**j**) and glucose production content of AML12 cells (**n**) with the indicated treatments were measured, as well as the expression levels of IL-6, TNF-α, and IL-1β (**k**–**m** and **o**–**q**). Experiments were performed at least in triplicate, and the results are shown as the mean ± s.d. Student’s *t*-test was used to analyze the data. (*P < 0.05; **P < 0.01; ***P < 0.001)

To identify the targets of exosomal miR-1249-3p, two bioinformatics tools (miRDB and TargetScan) were used to predict a set of common target genes. Among these, we verified that SKI family transcriptional corepressor 1 (SKOR1) was a direct target of miR-1249-3p (Fig. 4a). First, we found that SKOR1 protein expression was downregulated by miR-1249-3p in 3T3-L1 adipocytes and AML12 cells (Fig. 4b). However, the levels of SKOR1 mRNA essentially did not change (Supplementary Fig. S4a, b). Subsequently, sequence alignment between miR-1249-3p and full-length SKOR1 showed that the 3′UTR of SKOR1 may be a potential target of miR-1249-3p (Fig. 4c). Then, wild-type and mutated miR-1249-3p-binding sites were cloned into luciferase vectors. We observed a marked decrease in luciferase activity in 3T3-L1 adipocytes that were co-transfected with the wild-type binding site vector in the presence of miR-1249-3p. While, cells containing the mutated binding site vector did not exhibit such repression (Fig. 4d). Moreover, the SKOR1 protein was remarkably upregulated in the VATs and livers of HFD mice (Supplementary Fig. S4c). However, there was no change of SKOR1 expression at the mRNA level (Supplementary Fig. S4d). In addition, NCD-Exos decreased SKOR1 protein expression in the VATs and livers of HFD mice (Fig. 4e). These results reveal that SKOR1 is a direct target of miR-1249-3p in adipocytes and hepatocytes.

To determine the function of SKOR1 in regulating glucose homeostasis, we knocked down SKOR1 expression with short hairpin RNAs (shRNAs) in 3T3-L1 adipocytes and AML12 cells, and the effect was assessed by qRT-PCR and immunoblotting analyses (Supplementary Fig. S4e, g). We found that knockdown of SKOR1 increased insulin-stimulated glucose uptake in 3T3-L1 adipocytes and promoted the suppressive effect of insulin on glucose production in AML12 cells, along with reduced pro-inflammatory factors in both cells (Fig. 4f–i), which was neutralized by miR-1249-3p inhibitor (Fig. 4j–q). Similarly, overexpression of SKOR1 (Supplementary Fig. S4f, g) induced insulin resistance and inflammation in 3T3-L1 adipocytes and AML12 cells (Supplementary Fig. S5a–d), which was neutralized by the miR-1249-3p mimic (Supplementary Fig. S5e–l). Taken together, these findings suggest that SKOR1 is a direct downstream target of miR-1249-3p and mediates glucose homeostasis.

### SKOR1 interacts with SMAD6 in adipocytes and hepatocytes

**Fig. 5:**
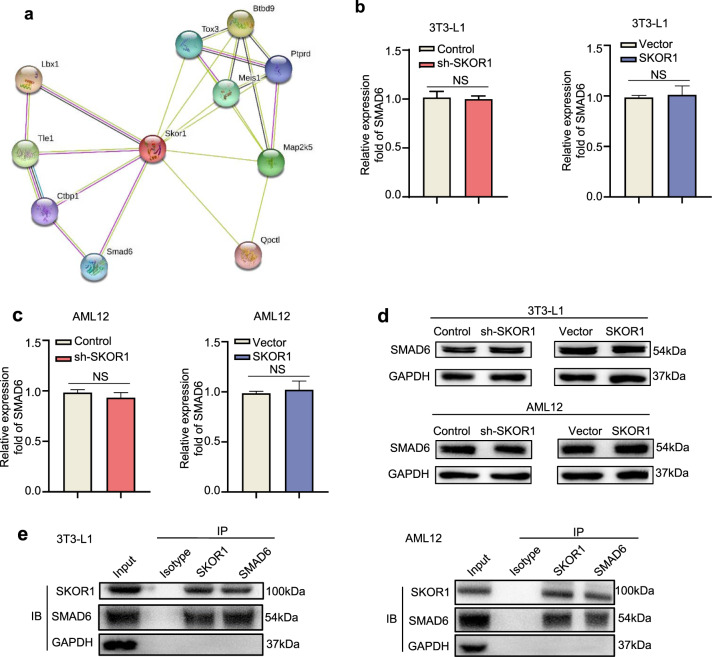
SKOR1 interacts with SMAD6 in adipocytes and hepatocytes. **a** Protein interaction prediction map of SKOR1. **b**, **c** The effect of sh-SKOR1 or Flag-SKOR1 on SMAD6 gene expression in 3T3-L1 adipocytes (**b**) and AML12 cells (**c**). **d** Western blot analysis of SMAD6 expression in 3T3-L1 adipocytes and AML12 cells with SKOR1 knockdown or overexpression. **e** Co-IP experiment of SKOR1 and SMAD6 in 3T3-L1 adipocytes and AML12 cells. Anti-SKOR1 and Anti-SMAD6 reciprocal IPs were blotted with antibodies against SKOR1 and SMAD6. Experiments were performed at least in triplicate, and the results are shown as the mean ± s.d. Student’s *t*-test was used to analyze the data. (*P < 0.05; **P < 0.01; ***P < 0.001)

To further explore how SKOR1 participates in the regulation of T2D, a protein interaction prediction tool (http://string-db.org) was used to assess that SKOR1 interacts with multiple proteins, such as ladybird homeobox 1 (LBX1) and SMAD family member 6 (SMAD6) (Fig. 5a). Previous studies have shown that SMAD6 binds to Smad ubiquitination regulatory factor 1 (SMURF1) and Myeloid differentiation factor 88 (MyD88) to exert anti-inflammatory effects *via* the TLR4/NF-κB pathway.^32^ MyD88 is a key linker molecule in the TLR4 signaling pathway and is related to inflammation *via* the NF-κB pathway.^33^ Thus, we further explored the relationship between SKOR1 and SMAD6. Since SKOR1 does not change the expression of SMAD6 in 3T3-L1 adipocytes and AML12 cells (Fig. 5b–d), we assumed that SKOR1 could interact with SMAD6 to regulate inflammation *via* the TLR4/NF-κB pathway. Immunoprecipitation (IP) on SKOR1 protein with lysates of 3T3-L1 adipocytes and AML12 cells was performed and SMAD6 was detected in the SKOR1-IP from cell lysate (Fig. 5e). Then, reciprocal IP with anti-SMAD6 antibody was performed, and SKOR1 was detected in the SMAD6-IP fraction (Fig. 5e). Collectively, these results indicate that SKOR1 interacts with SMAD6 in adipocytes and hepatocytes.

### MiR-1249-3p relieves insulin resistance and inflammation *via* the SKOR1-SMAD6-TLR4-NF-κB axis

**Fig. 6:**
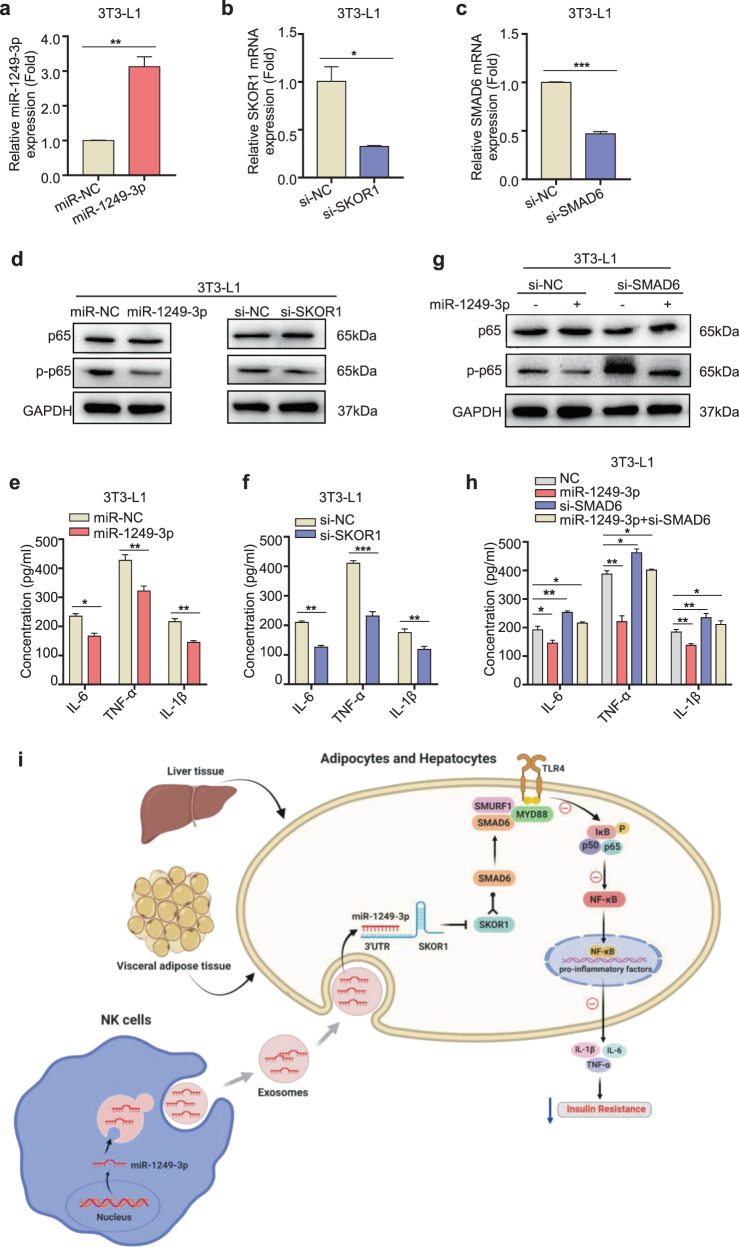
MiR-1249-3p relieves insulin resistance and inflammation *via* the SKOR1-SMAD6-TLR4-NF-κB axis. **a**–**c** After transfection with a miR-1249-3p mimic, miR-NC, or specific siRNA to SKOR1 or SMAD6, qRT-PCR analysis of miR-1249-3p (**a**), SKOR1 (**b**) and SMAD6 (**c**) expression in 3T3-L1 adipocytes cells with the indicated treatments was performed. **d**, **g** The expression of p-p65 and p65 in 3T3-L1 adipocytes cells with the indicated treatments was performed by western blot. **e**, **f**, **h** IL-1β, IL-6, and TNF-α expression in 3T3-L1 adipocytes cells subjected to the indicated treatments was assessed by ELISA. **i** The signaling pathway through which NK-derived exosomal miR-1249-3p regulates insulin resistance in type 2 diabetes mice. Experiments were performed at least in triplicate, and the results are shown as the mean ± s.d. Student’s *t*-test was used to analyze the data. (*P < 0.05; **P < 0.01; ***P < 0.001)

The above results showed that miR-1249-3p inhibits the expression of pro-inflammatory factors, such as IL-1β, IL-6, and TNF-α in 3T3-L1 adipocytes and AML12 cells, which are well-known targets of NF-κB signaling. Our study has proven that SKOR1 is a direct target of miR-1249-3p, and SKOR1 interacts with SMAD6. Previous studies have provided evidence that SMAD6 binds to SMURF1 and MyD88 to form the ternary complex SMAD6/MYD88/SMURF1, subsequently inhibiting TLR4 signaling.^32^ Furthermore, TLR4 binds to the corresponding ligand and further activates NF-κB, which promotes the upregulation of various inflammatory cytokines in a MyD88-dependent manner.^34^ Thus, we hypothesize that miR-1249-3p/SKOR1 axis play crucial role in the TLR4/NF-κB-dependent pro-inflammatory signaling. First, we transfected with a miR-1249-3p mimic or specific SKOR1 small interfering RNA (siRNA) into 3T3-L1 adipocytes and AML12 cells, respectively (Fig. 6a, b and Supplementary Fig. S6a, b). Immunoblot analysis and ELISA assay (Fig. 6d–f and Supplementary Fig. S6d–f) revealed that miR-1249-3p mimic or SKOR1 knockdown significantly inhibited the phosphorylation of p65, which subsequently downregulated the expression levels of pro-inflammatory genes, including IL-1β, IL-6, and TNF-α. These data indicate that the miR-1249-3p/SKOR1 axis inhibits pro-inflammatory gene expression by mediating NF-κB signaling suppression.

Since the role of SMAD6 in T2D has not been investigated, we for the first time reveals that SKOR1 functions by interacting with SMAD6 in T2D. To study whether SMAD6 is essential in miR-1249-3p mediated attenuated insulin resistance and inflammation, single miR-1249-3p mimic or combined with SMAD6 silencing was transfected into 3T3-L1 adipocytes and AML12 cells, respectively. The phosphorylation of p65 and pro-inflammatory factors levels were suppressed by miR-1249-3p overexpression or increased by SMAD6 silencing. Moreover, the inhibited effect of miR-1249-3p on TLR4/NF-κB axis activation is relieved, but not completely blocked in the presence of SMAD6 silencing (Fig. 6c, g, h and Supplementary Fig. S6c, g, h), suggesting that SMAD6 is essential to miR-1249-3p-mediated attenuated insulin resistance and inflammation, and other signaling pathways may exist in this process. Collectively, these results suggest that NK-derived exosomal miR-1249-3p alleviates insulin resistance and inflammation *via* the SKOR1-SMAD6-TLR4-NF-κB axis (Fig. 6i).

## Discussion

Components of the innate immune system including NK cells play a crucial role in regulating metabolic homeostasis.^35^ In fact, researchers reported that NK cell activity is significantly lower in patients with T2D and deteriorates linearly with the degree of hyperglycemia, which in turn disrupts immune system maintenance.^36^ In addition, NK cells have been reported as key regulators of obesity and insulin resistance in situ, such as in VATs and epididymal fat.^37,38^ Adipose NK cells possibly control macrophages as an upstream regulator by producing proinflammatory mediators, including IFN-γ, TNF-α, or IL-6, thereby contributing to the development of obesity-induced insulin resistance.^15,17,39^ According to Fig. 1f, there was no significant difference in the number of exosomes secreted by splenic NK cells between NCD mice and HFD mice. However, miR-1249-3p was highly expressed in splenic NK cells, NK-derived exosomes, and circulating exosomes from NCD mice than HFD mice (Fig. 3b), indicating that exosomes secreted by splenic NK cells can be absorbed by distal recipient cells *via* entry into the circulatory system. In our study, we explored that splenic NK cells regulate insulin resistance by secreting exosomes, which are then transferred to distant VATs and livers, rather than in situ.

Mechanism research reveals that NK-derived exosomal miR-1249-3p relieves insulin resistance and inflammation by targeting SKOR1, which interacts with SMAD6 and promotes glucose homeostasis by suppressing the TLR4/NF-κB signaling pathway. As detailed, miR-1249-3p is overexpressed in NK-derived exosomes from lean mice and negatively regulates the expression of SKOR1 in adipocytes and hepatocytes. In addition, MYD88 and SKOR1 are competitively combined with SMAD6. As SKOR1 is downregulated by miR-1249-3p, more MYD88 binds with SMAD6 and SMURF1 to form ternary complexes, which inhibit the release of pro-inflammatory cytokines by suppressing the TLR4/NF-κB signaling pathway. Thus, NK-derived exosomal miR-1249-3p attenuates insulin resistance and inflammation to promote glucose homeostasis in mouse models of T2D.

As shown in Fig. 4a, miR-1249-3p is predicted to target SKOR1 and SMPD3. SMPD3, which encodes nSMase2, hydrolyzes sphingomyelin to release ceramide and phosphorylcholine. The expression of SMPD3 is significantly increased in high liver fat individuals^40^ and contributes to the increase in ceramide content,^41^ which leads to adverse effects due to long-chain fatty acids on insulin resistance and inflammation. Moreover, researchers have reported that ceramide is elevated in the plasma of obese patients with T2D,^42^ and chronic elevation of tissue ceramides leads to persistent impairment of metabolic homeostasis, thereby driving insulin resistance and hepatic steatosis.^43^ Reasonably, the regulatory effect of miR-1249-3p on SMPD3 in T2D requires further investigation.

SKOR1 has been widely reported to play a transcriptional regulatory role on genes related to restless legs syndrome,^44^ but is new to T2D. This prompted us to explore the relationship between SKOR1 and T2D. Herein, we prove that SKOR1 is the target of NK-derived exosomal miR-1249-3p and destroys glucose homeostasis *via* the TLR4/NF-κB pathway. In addition, previous studies report that SMAD6 is highly expressed in pancreatic cancers and enhances anchorage-independent growth in pancreatic cancer cells by blocking TGF-β-mediated growth inhibition^45^. SMAD6 supports lung cancer cell growth as well as breast cancer cell invasion, resulting in reduced patient survival^46,47^. However, the role of SMAD6 in T2D has not been investigated to date. Our study reveals for the first time that SKOR1 functions by interacting with SMAD6 in T2D. Nevertheless, SKOR1 is predicted to interact with a series of proteins such as LBX1 and TOX3 (Fig. 5a), whose relationship with T2D remains unclear and is thus worth investigating.

In summary, this study reveals that NK-derived exosomal miR-1249-3p attenuates insulin resistance and inflammation through the SKOR1-SMAD6-TLR4-NF-κB axis, and provides a series of potential therapeutic targets in T2D. Future studies are warranted to determine the therapeutic effect of miR-1249-3p and SKOR1 blocker in T2D mice and clinical trials.

## Materials and methods

### Animal study

Approximately 120 six-week-old male C57B/6 mice were purchased from Nanjing Biomedical Research Institute of Nanjing University (Nanjing, China). Five mice per cage were housed in atmospheric conditions that were controlled at an ambient temperature of 20 ± 2 °C, a humidity of 45–55%, and a light/darkness cycle of 12/12 h. Food and tap water were supplied ad libitum during the entire protocol. After an acclimation period of 7 days, during which they were fed normal chow (10% fat calories, 20% protein calories, and 70% carbohydrates; Biotech-hd, Beijing, China), the mice were randomly assigned to two diet groups: NCD mice (*n* = 48) and HFD mice (*n* = 72). At the time of randomization, the mice weighed approximately 21 g. The procedures of all animal experiments were complied with Institutional Animal Care and Use Committee (IACUC) regulations. All animal experiments were approved by the Ethics Committee of China Pharmaceutical University. Permit Number: SYXK2016-0011.

### Animal models

Upon initiation of the group diet, NCD mice were fed a normal chow diet, and HFD mice were fed a high fat diet (45% fat calories, 20% protein calories, and 35% carbohydrate; Biotech-hd, Beijing, China) during the rest of the protocol. During this period, the body weight and blood glucose of mice were recorded weekly. Thereafter, HFD mice were injected with STZ (40 mg/kg) at 35, 36, 37 days after the HFD began. The OGTT and ITT of HFD mice and NCD mice were performed 42 days after the initiation of the group diet. Twenty NCD mice and 20 HFD mice were euthanized at 45 days by exsanguination after ketamine and xylazine anesthesia for spleen NK cell extraction. Liver, VATs, and serum samples were collected for future analysis. The schematic diagram of group treatment and index detection of NCD and HFD mice are shown in Supplementary Fig. S1a.

### Cell culture

The 3T3-L1 adipocytes and AML12 cells were purchased from Saihongrui (Nanjing, China). 3T3-L1 cells were induced into mature 3T3-L1 adipocytes using induction medium A (high-glucose DMEM medium with 10% FBS, 1 μM dexamethasone, 0.5 mM 3-isobutyl-1-methylxanthine, and 10 μg/mL insulin) for 2 days and induction medium B (high-glucose DMEM medium with 10% FBS, and 10 μg/mL insulin) for 2 days, and finally cultured in DMEM medium (hyclone, Los Angeles, USA) with 10% FBS (Biological Industries, Israel). AML12 cells are mouse hepatocytes that were cultured in 1:1 mixed DMEM/F12 (Invitrogen, California, USA) supplemented with 1% ITS liquid media (Sigma-Aldrich, Missouri, USA), dexamethasone 40 ng/mL, and 10% FBS. All of the cell lines were maintained in a 37 °C humidified incubator with 5% CO_2_. Their species origins were verified with PCR, and the identities of the cell lines were verified by short tandem repeat (STR) profiling. Both cell lines were checked for mycoplasma using a MycoAlert Mycoplasma Detection Kit (Lonza, Valais, Switzerland).

### Antibodies

Antibodies for CD3 (100243), CD9, (124802) and CD49b (103501) antibodies were purchased from BioLegend (San Diego, California, USA). Antibodies against HSP70 (RK-200-301-A27) were purchased from Lianke (Hangzhou, China). Antibodies specific to CD63 (WL02549), GRP94 (WL04446), TSG101 (WL05130), Akt (WL0003b), pAkt (WLP001a), PPARγ (WL01800), GLUT4 (WL02425), NK-κB p65 (WL01273b), and p-NK-κB p65 (WL02169) were purchased from Wanleibio (Shenyang, China), antibody specific to SMAD6 (ab273106) was purchased from abcam (Shanghai, China), the details of the all reagents and kits were listed in Supplementary Table S2.

### Western blotting

Tissues, cells, and exosome lysates were homogenized in RIPA buffer (KeyGEN BioTECH, Nanjing, China) and quantified using bicinchoninic acid (BCA) assay (KeyGEN BioTECH, Nanjing, China). Protein samples were separated by electrophoresis and followed by blocking with 5% non-fat milk in TBST (Tris-buffered saline with Tween-20 solution) for 2 h at room temperature. Next, PVDF membranes with indicated protein samples were incubated with diluted primary antibodies at 4 °C overnight. After washing with TBST for 5 × 10 min, PVDF membranes with indicated protein samples were directly incubated with diluted secondary antibodies at room temperature for 1 h. After washing with TBST for 5 × 10 min, chemiluminescence signals were detected using BeyoECL Plus (Beyotime, Shanghai, China). Protein expression was normalized to GAPDH levels.

### NK cell isolation and culture

NK cells were isolated from splenic lymphocytes of NCD and HFD mice. Spleens were separated and passed through a 70 μm cell strainer, splenocytes were treated with red blood cell lysis buffer in an ice bath for 5 min. Then, lymphocytes were concentrated by centrifugation at 1,200 rpm for 5 min and resuspended in RPMI 1640 medium. Single cell suspensions of lymphocytes were incubated with fluorescence-tagged antibodies against CD3 or CD49b (DX5). CD3^−^CD49b^+^ NK cells were purified using a BD FACS Aria flow cytometer (BD Biosciences, New York, USA). NK cells were then cultured in RPMI 1640 medium with 10% exosome-free FBS to produce exosomes.

### Isolation and analysis of exosomes

For exosome isolation from cells, NK cells were cultured in T75 cm^2^ flasks for 48 h in RPMI 1640 medium containing 10% exosome-free FBS. The culture medium (CM) was collected and filtered through 0.22 μm filters (Millipore, Massachusetts, USA). Exosomes in CM were isolated by ultracentrifugation according to the standard method. First, samples were centrifuged at 10,000×*g* for 10 min to remove other cellular debris, then the supernatant was collected and centrifuged at 100,000×*g* for 70 min to pellet exosomes. Ultracentrifugation experiments were performed with Optima L-80XP (Beckman Coulter, Florida, USA). For exosome isolation from serum, mouse blood was collected in 1.5 mL tubes and allowed to clot for 1 h at room temperature, and then serum was obtained by centrifugation at 2000×*g* for 10 min at 4 °C. Then, exosomes were isolated from serum by ultracentrifugation. Bicinchoninic acid (BCA) assay was performed to measure protein concentrations in exosomes. For nanoparticle tracking analysis, the size and number of exosomes were detected by ZetaView (Microtrac, USA). For transmission electron microscopy observation, 10 μg of exosomes was dropped onto carbon-coated copper grids and baked for 5 min under infrared light. Exosome samples were then negatively stained with 3% (w/v) aqueous phosphotungstic acid for 5 min. The embedded samples were observed under an HT7700 transmission electron microscope (Hitachi, Tokyo, Japan).

### Exosome tracing

First, NCD-Exos were labeled using PKH26 Red Fluorescent Cell Linker Kits (Sigma-Aldrich, Missouri, USA) according to the manufacturer’s instructions and collected by ultracentrifugation at 100,000×*g* for 70 min. Then, 10 μg of PKH26-labeled NCD-Exos were adoptively transferred into recipient mice *via* tail vein injection. Finally, 24 h after injection, various tissues were harvested (livers, islets, subcutaneous adipose tissues, visceral adipose tissues, and skeletal muscle tissues) for ex vivo imaging.

### In vivo and in vitro exosome treatment

For in vivo treatment, nine HFD mice were randomly divided into three groups: G1 (HFD + Control Liposomes), G2 (HFD + HFD-Exos) and G3 (HFD + NCD-Exos). Nine NCD mice were randomly divided into three groups: G4 (NCD + Control Liposomes), G5 (NCD + HFD-Exos) and G6 (NCD + NCD-Exos) groups. Ten microgram of exosomes was adoptively transferred into recipient mice *via* tail vein injection at 50, 52, and 54 days after group feeding. Blank liposomes (FormuMax, California, USA) was diluted 100 times before injection and used as control. Thereafter, the blood glucose of each group was recorded weekly. OGTT, ITT, GHb (glycated hemoglobin), GSP (glycated serum protein) of the HFD and NCD mice were measured after exosome treatment. Then, the mice were euthanized by exsanguination after ketamine and xylazine anesthesia. The liver and VAT samples of the mice were collected for further analysis.

For in vitro treatment, 2 μg of NK cells-derived exosomes was added to the culture media of 3T3-L1 adipocytes for glucose uptake assay or AML12 cells for glucose output assay. Then, the cells were collected for protein extraction and analysis of phosphorylated Akt, PPARγ, and GLUT4.

### Oral glucose tolerance test (OGTT)

After 16 h of fasting, blood glucose was measured in conscious mice at 0, 30, 60, 90, and 120 min after the administration of 1 g/kg glucose by oral gavage using the Sannuo blood glucose monitor (Sinocare, Hunan, China).

### Insulin tolerance test (ITT)

Approximately 0.5 U/kg insulin was intraperitoneally injected into mice that were fasted for 6 h. Blood glucose levels were subsequently determined at 0, 30, 60, and 120 min after injection, and the area under the curve (AUC) of blood glucose *versus* time was calculated.

### Determination of GHb (glycated hemoglobin)

Mice were fasted overnight. Whole blood was collected in an anticoagulation tube and gently inverted to mix well. Red blood cells were isolated by centrifugation at 1000 rpm for 10 min, and then washed with normal saline. The supernatant was collected for the GSP assay. Then, 1 mL of hematocrit blood was added to 1.5 mL of pre-cooled double distilled water and mixed thoroughly to prepare lysed blood. GHb was tested using the glycated hemoglobin kit (Nanjing Jiancheng Bioengineering Institute, Nanjing, China). GHb indication = (Measured OD value − Blank OD value)/Grams of hemoglobin in 2 mL of hemolyzed blood × Dilution factor × 10 g.

### Determination of GSp (glycated serum protein)

The supernatant obtained from the above anticoagulated whole blood was centrifuged at 9000 rpm for 15 min, then the plasma was collected from the supernatant. GSP was detected using the glycated serum protein detection kit (Nanjing Jiancheng Bioengineering Institute, Nanjing, China). GSP content (mmol/L) = Measured OD value/(Standard OD value − Standard blank OD value) × Standard concentration 2 mmol/L.

### In vivo insulin-stimulated Akt phosphorylation assay

Insulin action in tissues was measured by insulin-stimulated Akt phosphorylation in the livers and VATs. After 6 h of fasting, mice were injected with insulin (0.7 U/kg body weight) *via* intraperitoneal injection. Then parts of the liver and VAT were collected after 30 min for the detection of phosphorylation Akt using western blot analysis.

### Hepatic TG analysis

The TG concentrations in liver tissue samples were determined using a triglyceride (TG) assay kit (mlbio, Shanghai, China) according to the manufacturer’s protocol.

### Glucose uptake assay

After stimulating with 100 nM insulin for 24 h, 3T3-L1 adipocytes were cultured at 37 °C for 2 h with glucose uptake buffer (no phenol red, glucose-free DMEM medium containing 0.2 M glucose), then the cell culture supernatant was collected for determination of glucose content using a glucose detection kit (Nanjing Jiancheng Bioengineering Institute, Nanjing, China).

### Glucose output assay

After 12 h serum starvation, AML12 cells were stimulated with 10 nM insulin at 37 °C for 24 h with glucose production buffer (no phenol red, glucose-free DMEM medium containing 20 mM l-sodium lactate, 2 mM sodium pyruvate, 2 mM l-glutamine, and 15 mM N-2-hydroxyethylpiperazine-N-ethane-sulphonicacid) (Sangon biotech, Shanghai, China), and then the cell culture supernatant was collected for determination of glucose content using a glucose detection kit.

### Microarray analysis of exosomal miRNAs

Exosomal miRNA microarray analysis was performed at CapitalBio Technology, Inc. (Beijing, China) using Agilent Mouse miRNA 8 × 60K V21.0 Microarray (Agilent Technologies, California, USA). Quantile normalization and subsequent data processing were performed using Quantile algorithm, GeneSpringGX Software (Agilent Technologies, California, USA). Hierarchical clustering analysis of miRNA differential expression was performed using Euclidean distance analysis with Morpheus Online Software.

### Luciferase reporter assays

For the identification of the binding sites between miR-1249-3p and SKOR1, cells were transfected with a luciferase construct containing SKOR1 with the wild-type or a mutated version of the binding site and co-transfected with a miR-1249-3p mimic or empty vector. To evaluate the luciferase activity of SKOR1, cells were transfected with a luciferase construct containing SKOR1 with the wild-type or a mutated version of the binding site. The luciferase vectors were constructed by GenePharma Co. (Shanghai, China). After transfection, a dual luciferase kit (Promega, USA) was used to detect the luciferase activities according to the manufacturer’s instructions.

### Gene knockdown and overexpression

For transient transfection, 3T3-L1 adipocytes and AML12 cells were transfected with miR-1249-3p mimic, miR-NC, inhibitor, inh-NC, or siRNAs of SKOR1, SMAD6 (Genepharma, Shanghai, China) respectively, and their sequences are listed in Supplementary Table S3. For SKOR1 knockdown, 3T3-L1 adipocytes and AML12 cells were transfected with short hairpin RNAs (Genepharma, Shanghai, China) targeting SKOR1 and negative control RNA duplex (Genepharma, Shanghai, China) using lipofectamine 3000 (Invitrogen, California, USA) according to the manufacturer’s instructions. For SKOR1 overexpression, Flag-SKOR1 (Genepharma, Shanghai, China) were transfected into 3T3-L1 adipocytes and AML12 cells using Lipofectamine 3000 (Invitrogen, California, USA) according to the manufacturer’s instructions.

### RNA isolation and qRT-PCR

Total RNA was extracted from cells or tissues using TRIzol reagent (Invitrogen, California, USA) according to the manufacturer’s instructions. For mRNA detection, reverse transcription was performed using HiScript III RT SuperMix (Vazyme, Nanjing, China). Quantitative relative real-time PCR (qRT-PCR) was measured using ChamQ Universal SYBR qPCR Master Mix (Vazyme, Nanjing, China) and performed on Thermo Fisher QuantStudio 3 (Thermo Fisher Scientific, Shanghai, China). β-Actin was used as reference control. For miRNA analysis, poly (A) addition to miRNA and reverse transcription were performed using a miRNA cDNA synthesis kit (abm, Zhenjiang, China). Quantitative relative real-time PCR was conducted using EvaGreen miRNA qPCR Master Mix (abm, Jiangsu, China) and performed on Thermo Fisher QuantStudio 3 (Thermo Fisher Scientific, Shanghai, China). U6 was used as reference control. The sequences of all indicated primers are listed in Supplementary Table S4.

### ELISA assays

IL-1β, IL-6, and TNF-α levels in the culture medium of 3T3-L1 adipocytes and AML12 cells were measured using an ELISA kit (Annuoruikang, TianJin) according to manufacturer’s guidelines.

### Co-immunoprecipitation

For immunoprecipitation studies, 1 × 10^8^ 3T3-L1 adipocytes or AML12 cells were cultured and washed twice with ice cold PBS. Total protein was collected using RIPA buffer (KeyGEN BioTECH, Nanjing, China) and quantified using bicinchoninic acid (BCA) assay. Co-IP was conducted using anti-SKOR1 (Invitrogen, California, USA, PA5-68897) or anti-mouse SMAD6 antibody (Santa Gruz Biotechnology, USA, sc25321). The immune complexes were captured on Protein A/G agarose beads (Beyotime, Shanghai, China). Samples were tumbled overnight at 4 °C for at least 12 h. Immunocomplexes were pulled down using Protein A Dynabeads from Invitrogen for 6 h at 4 °C. Then, the beads were washed thrice with ice-cold PBS and resuspended in 50 μL loading buffer. Samples were heated at 95 °C for 5 min and placed on ice for 1 min before immunoblotting. Twelve percentage of Tris-acetate gels were used in gel electrophoresis. Proteins were then transferred onto 0.45 μm PVDF membranes at 220 mA for 1 h. Membranes were blocked with 5% BSA, stained with primary antibody overnight at 4 °C, and stained with secondary antibody for 1 h at room temperature. Proteins were detected using BeyoECL Plus (Beyotime, Shanghai, China).

### Statistical analysis

Data analysis was performed using GraphPad prism v5.0. Each experiment was conducted in triplicate at the least and all results are presented as the mean ± SEM. Student’s *t*-test and two-way ANOVA test were used to assess statistical significance. Differences with a value of *P* < 0.05 were considered to be significant, whereas those with *P* < 0.01 were deemed to be highly significant.

## Supplementary information

Supplementary Materials

## Data Availability

The data sets used and/or analyzed during the current study are available from the corresponding author upon reasonable request.
