# Supplementary material for: Natural killer cell-derived exosomal miR-1249-3p attenuates insulin resistance and inflammation in mouse models of type 2 diabetes
Source: Signal Transduct Target Ther. 2021 Nov 30;6:409. doi: 10.1038/s41392-021-00805-y (PMC8632983; doi:10.1038/s41392-021-00805-y)
Supplement: Supplementary file 1 — Supplementary Materials [file 41392_2021_805_MOESM1_ESM.docx]

**Supplementary Materials for**

**Natural killer cell-derived exosomal miR-1249-3p attenuates insulin resistance and inflammation in mouse models of type 2 diabetes**

**Ying Wang^1,2,3^, Mengwei Li^1,2,3^,** Lin Chen^1,2^, Huan Bian^1,2^, Xiangying Chen^1,2^, Huilin Zheng^1,2^, Peiwei Yang^1,2^, Quan Chen^1,2^, **Hanmei Xu^1,2^**

Correspondence to: 13913925346@126.com

**This file includes:**

**Figures. S1 to S6**

**Tables S1 to S4**

**
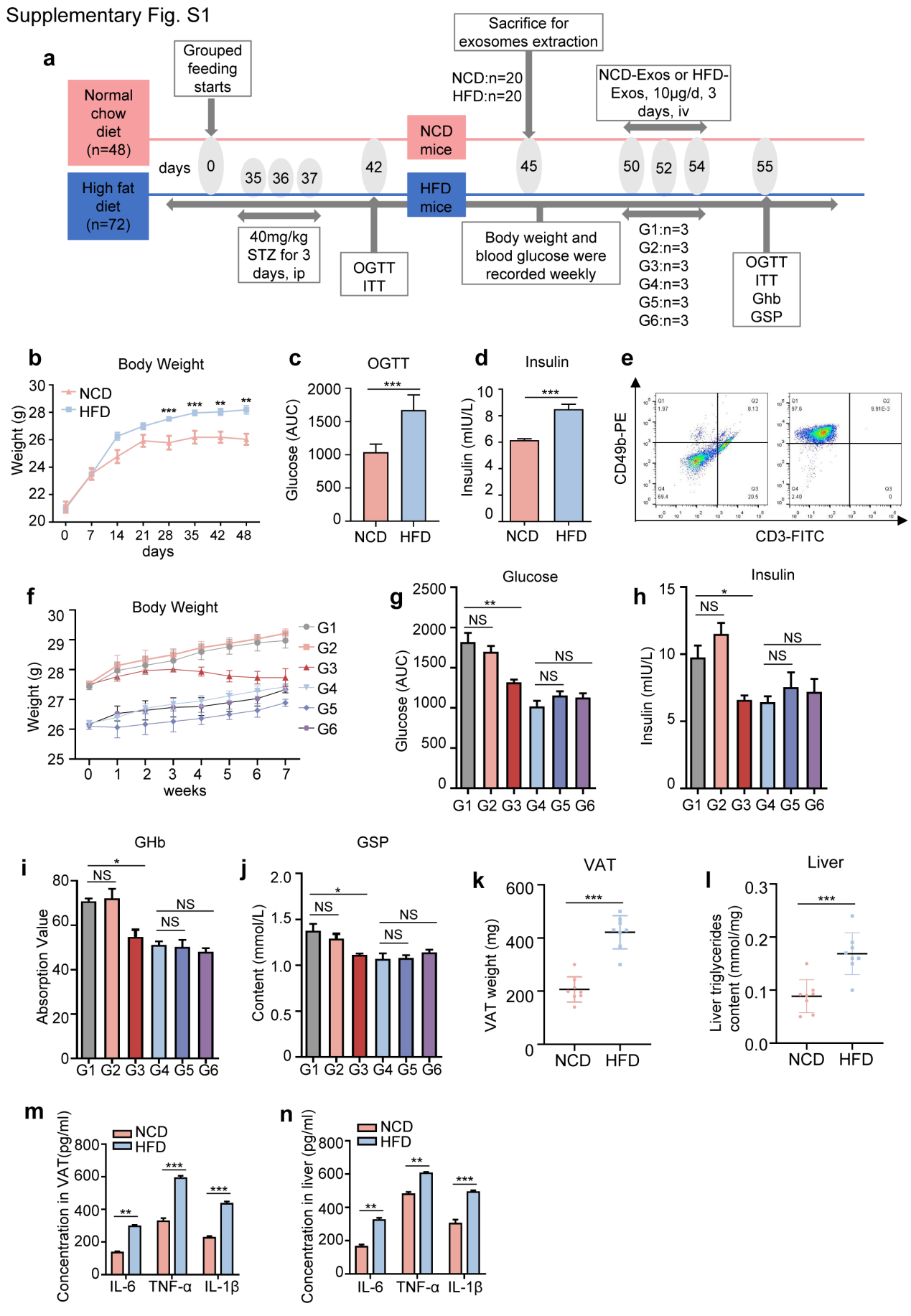
**

**Supplementary Fig. S1 NK-derived exosomes from lean mice attenuate obesity-induced insulin resistance.** **(a)** Schematic diagram of group treatment and index detection of NCD and HFD mice **(b)** After grouped feeding, the body weight of mice was recorded weekly. **(c-d)** The AUC of OGTT **(c)** and fasting insulin levels **(d)** of NCD mice (n = 48) and HFD mice (n = 72). **(e)** Left panel: flow sorting of mouse spleen NK cells. Right panel: purity identification of mouse spleen NK cells. The Q1 region is NK cells. **(f)** After blank liposomes, NCD-Exos and HFD-Exos were respectively injected into NCD and HFD mice, the body weight of mice was recorded weekly. G1: HFD mice treated with blank liposomes (n = 3); G2: HFD mice treated with HFD-Exos (n = 3); G3: HFD mice treated with NCD-Exos (n = 3); G4: NCD mice treated with blank liposomes (n = 3); G5: NCD mice treated with HFD-Exos (n = 3); G6: NCD mice treated with NCD-Exos (n = 3). **(g-j)** After blank liposomes, NCD-Exos and HFD-Exos were respectively injected into NCD and HFD mice, the AUC of fasting blood glucose **(g)**, fasting insulin levels **(h)**, glycated hemoglobin (GHb) **(i)** and glycated serum protein (GSP) **(j)** were tested in each group. **(k)** The weight of VATs from NCD mice and HFD mice. **(l)** Liver triglycerides content of NCD mice and HFD mice. **(m, n)** ELISA assays of IL-6, IL-1β, TNF-α expression in VATs (m) and livers (n) of NCD and HFD mice. Experiments were performed at least in triplicate and results are shown as mean ± s.d. Student’s t-test was used to analyze the data. (**P* < 0.05; ***P* < 0.01; ****P* < 0.001).

**
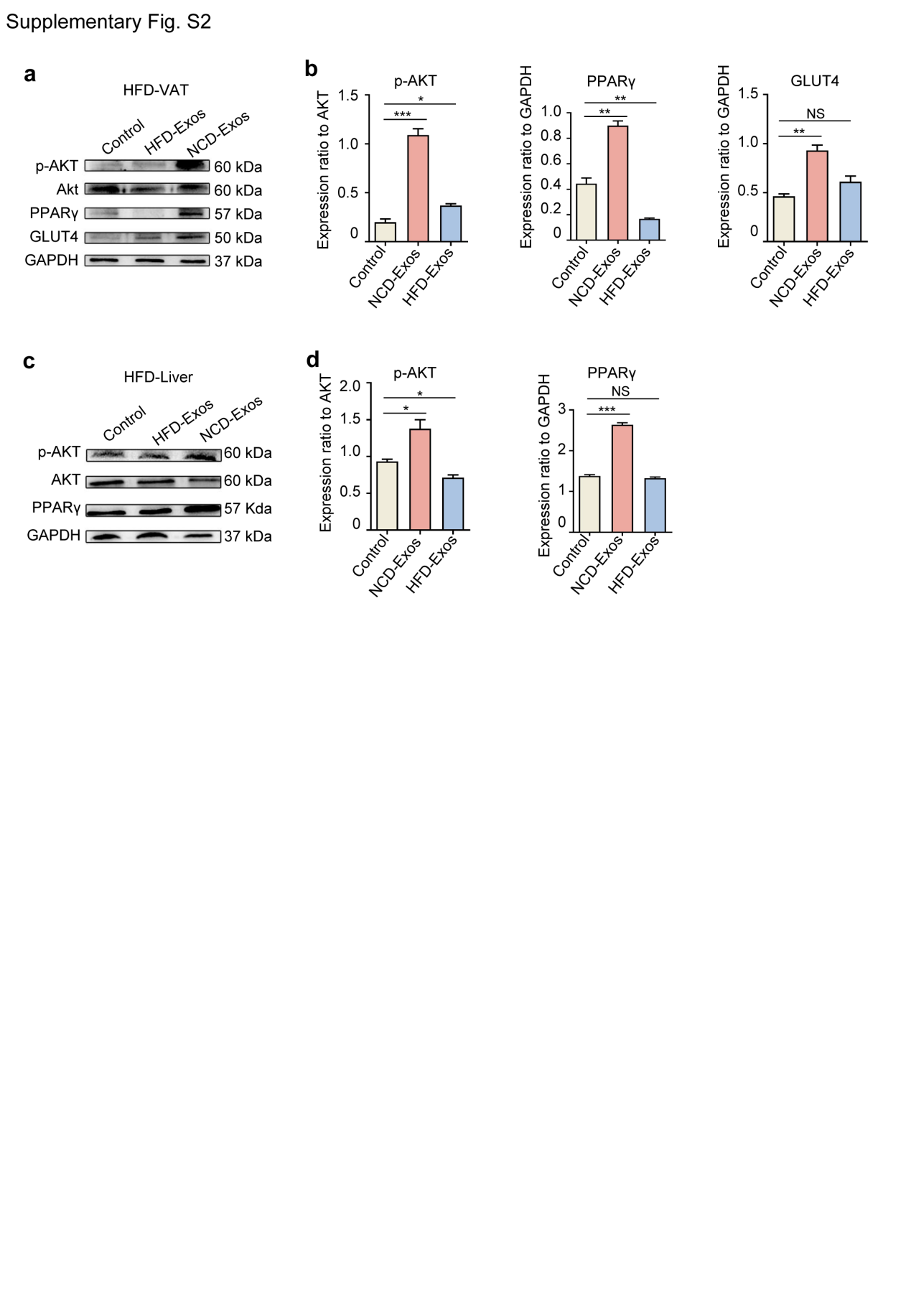
**

**Supplementary Fig. S2 NK-derived exosomes from lean mice enhance insulin sensitivity in obese mice. (a, b)** Western blot assays of p-Akt, Akt, PPARγ and GLUT4 expression in VATs of HFD mice that treated with NCD-Exos or HFD-Exos. **(c, d)** Western blot assays of p-Akt, Akt and PPARγ expression in livers of HFD mice that treated with NCD-Exos or HFD-Exos. Experiments were performed at least in triplicate and results are shown as mean ± s.d. Student’s t-test was used to analyze the data. (**P* < 0.05; ***P* < 0.01; ****P* < 0.001).

**
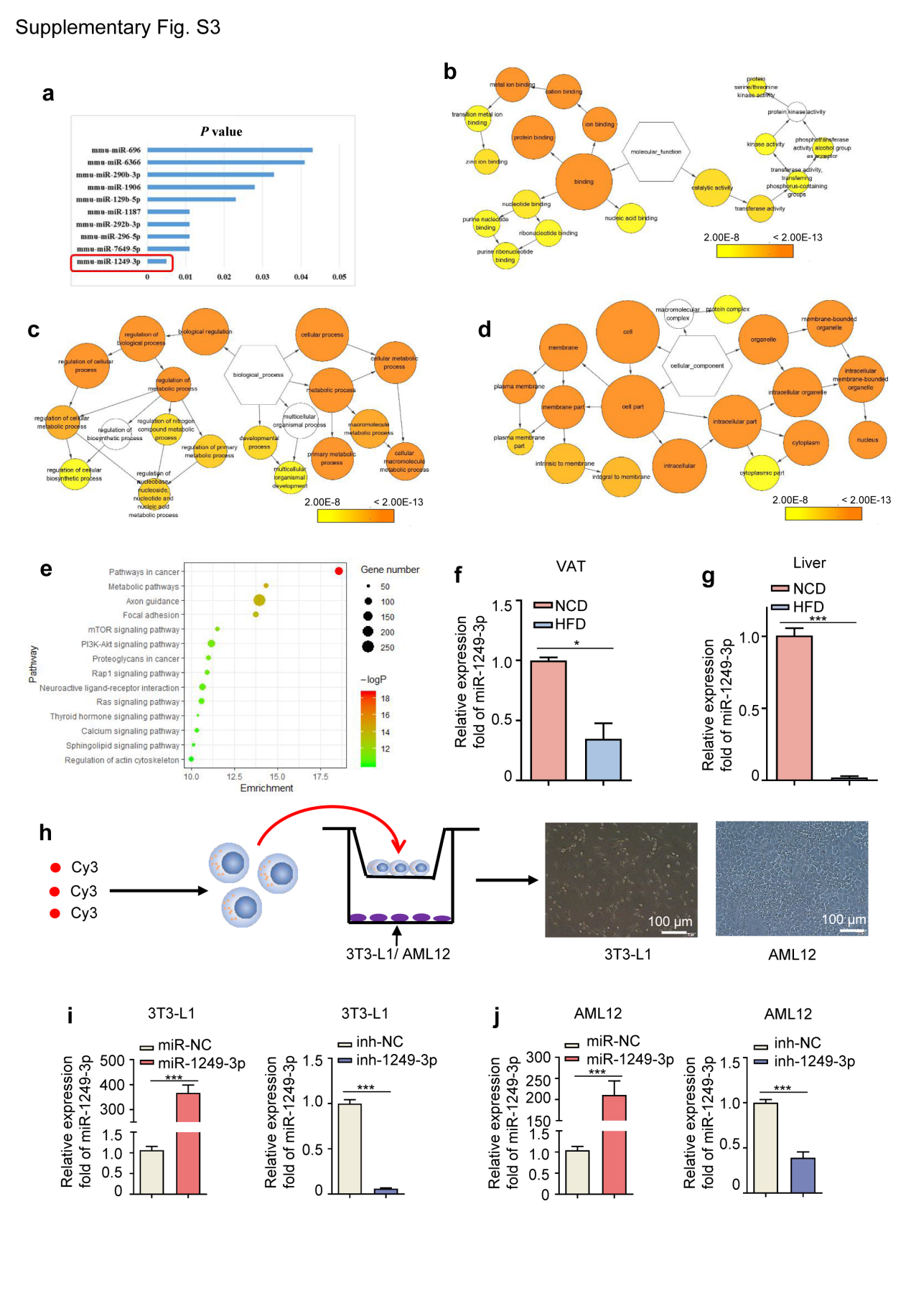
**

**Supplementary Fig. S3 NK-derived exosomal miR-1249-3p mediates cellular insulin sensitivity and inflammation.** **(a)** The P values of differentially expressed miRNAs were exhibited. **(b-d)** Biological process, cell component and molecular function hierarchical network diagrams of GO enrichment for differentially expressed miRNAs. **(e)** KEGG enrichment bubble diagram for differentially expressed miRNAs. **(f, g)** qRT-PCR assay of miR-1249-3p expression in livers **(f)** and VATs **(g)** of NCD and HFD mice. **(h)** In the control group, the appearance of Cy3 red fluorescence in 3T3-L1 adipocytes and AML12 cells was examined after co-cultured with NK cells that treated with Cy3 dye (without miR-1249-3p mimic) for 12 h. Scale bar, 100 μm. **(i, j)** MiR-NC, miR-1249-3p, inh-NC and inh-1249-3p were respectively transfected into 3T3-L1 adipocytes and AML12 hepatocytes. Experiments were performed at least in triplicate and results are shown as mean ± s.d. Student’s t-test was used to analyze the data. (**P* < 0.05; ***P* < 0.01; ****P* < 0.001).

**
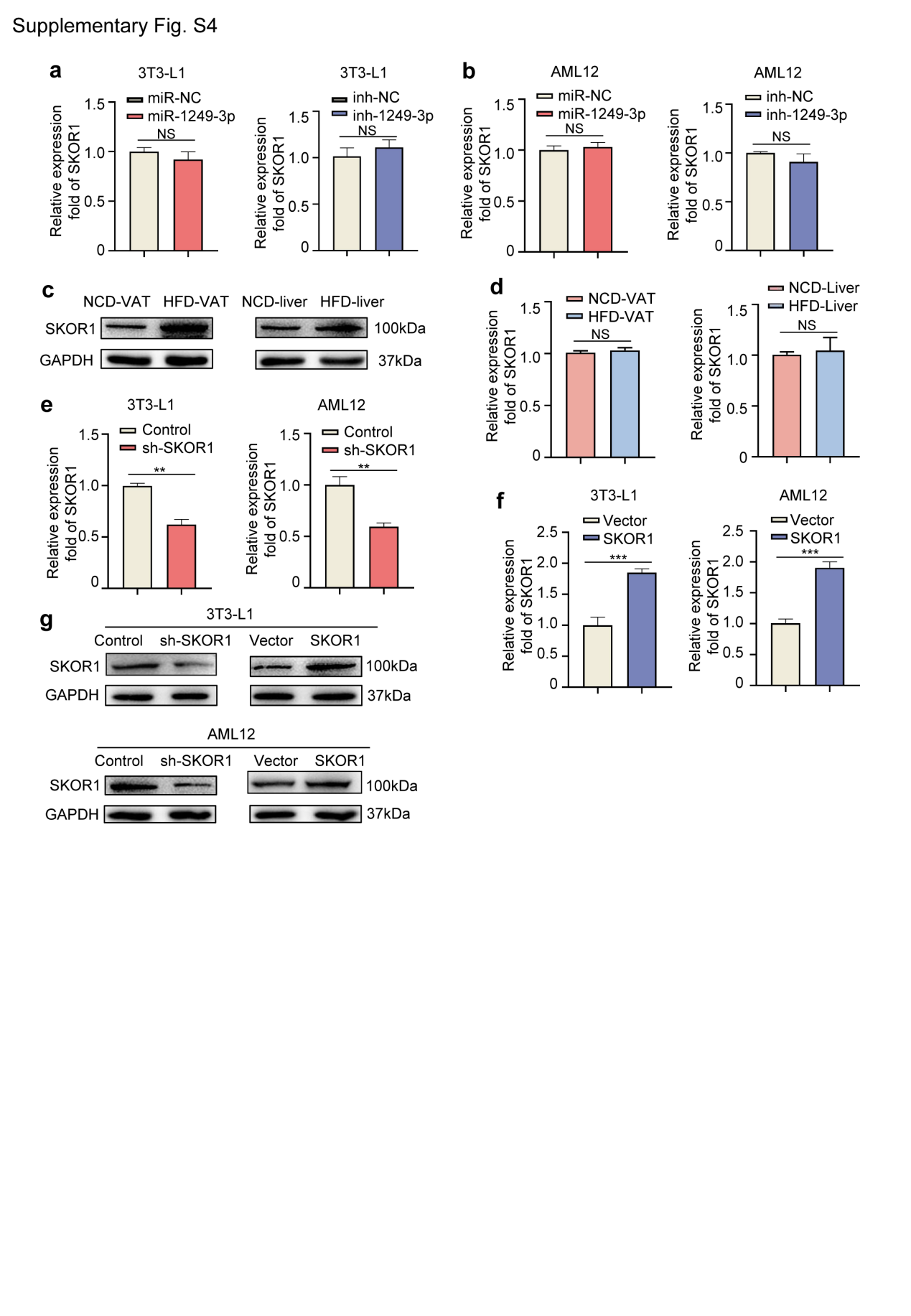
**

**Supplementary Fig. S4 Exosomal miR-1249-3p directly targets SKOR1 in adipocytes and hepatocytes. (a, b)** qRT-PCR assay of SKOR1 expression in 3T3-L1 adipocytes and AML12 cells with indicated treatments. **(c)** Western blot analysis of SKOR1 expression in VATs and livers of HFD mice and NCD mice. **(d)** qRT-PCR assay of SKOR1 expression in VATs and livers of NCD and HFD mice. **(e, f)** qRT-PCR assay of SKOR1 expression in 3T3-L1 adipocytes and AML12 cells with SKOR1 knockdown or overexpression. **(g)** Western blot assay of SKOR1 expression in 3T3-L1 adipocytes and AML12 cells with SKOR1 knockdown or overexpression. Experiments were performed at least in triplicate and results are shown as mean ± s.d. Student’s t-test was used to analyze the data. (**P* < 0.05; ***P* < 0.01; ****P* < 0.001).

**
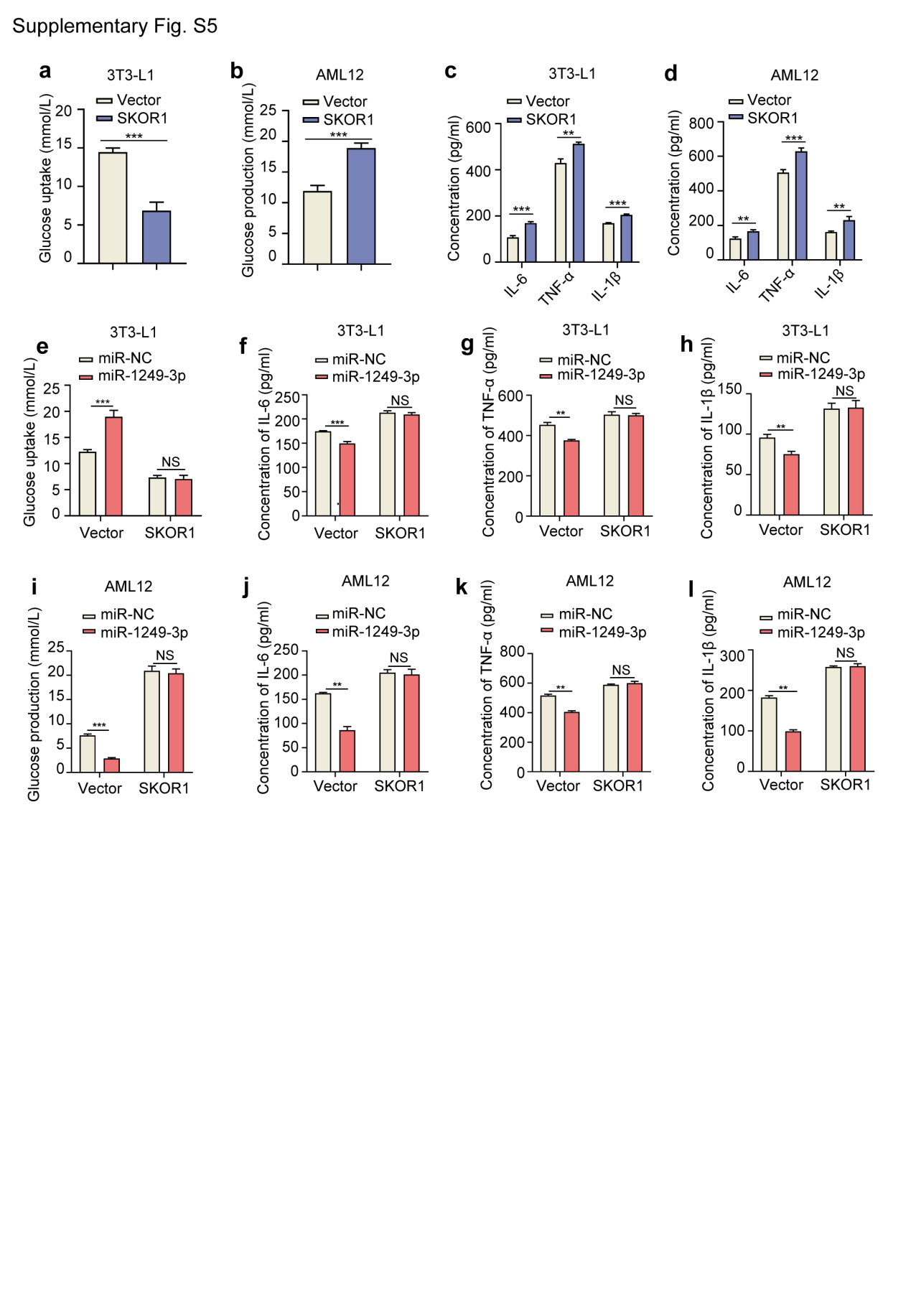
**

**Supplementary Fig. S5 Exosomal miR-1249-3p directly targets SKOR1 to mediate insulin sensitivity. (a-d)** The effect of Flag-SKOR1 on glucose uptake content of 3T3-L1 adipocytes **(a)**, glucose output content of AML12 cells **(b)**, and expression levels of IL-6, TNF-α and IL-1β **(c, d)**. **(e-l)** Glucose uptake content of 3T3-L1 adipocytes **(e)** and glucose production content of AML12 cells **(i)** with indicated treatments were measured, as well as expression levels of IL-6, TNF-α and IL-1β **(f-h and j-l)**. Experiments were performed at least in triplicate and results are shown as mean ± s.d. Student’s t-test was used to analyze the data. (**P* < 0.05; ***P* < 0.01; ****P* < 0.001).

**
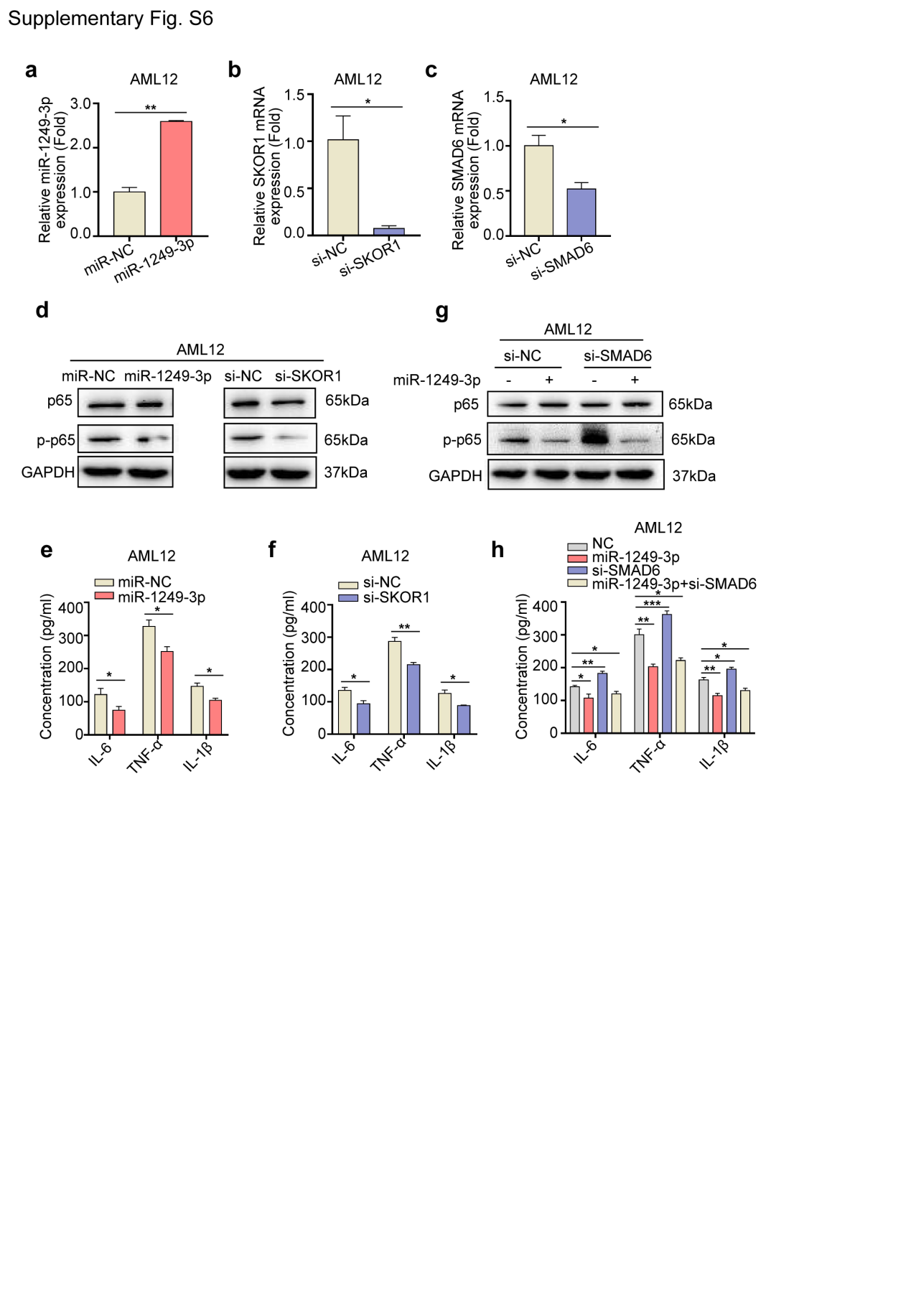
**

**Supplementary Fig. S6 MiR-1249-3p relieves insulin resistance and inflammation via the SKOR1-SMAD6-TLR4-NF-κB axis.** (**a-c**) After transfection with a miR-1249-3p mimic, miR-NC, or specific siRNA to SKOR1 or SMAD6, qRT-PCR analysis of miR-1249-3p (**a**), SKOR1 (**b**) and SMAD6 (**c**) expression in 3T3-L1 adipocytes cells with the indicated treatments was performed. (**d, g**) The expression of p-p65 and p65 in 3T3-L1 adipocytes cells with the indicated treatments was performed by western blot. (**e**, **f**, **h**) IL-1β, IL-6, and TNF-α expression in 3T3-L1 adipocytes cells subjected to the indicated treatments was assessed by ELISA. Experiments were performed at least in triplicate and results are shown as mean ± s.d. Student’s t-test was used to analyze the data. (**P* < 0.05; ***P* < 0.01; ****P* < 0.001).

**Supplementary Table S1. Differential expression analysis of miRNAs abundance between NCD-Exos and HFD-Exos.**

| Name | Fold Change ([H]vs[N]) | Relative expression | *P* value |
| --- | --- | --- | --- |
| mmu-miR-6366 | 10.826 | down | 0.041 |
| mmu-miR-1249-3p | 4.436 | down | 0.005 |
| mmu-miR-296-5p | 4.096 | down | 0.011 |
| mmu-miR-1906 | 3.369 | up | 0.028 |
| mmu-miR-7649-5p | 2.612 | up | 0.011 |
| mmu-miR-696 | 2.578 | up | 0.043 |
| mmu-miR-292b-3p | 2.350 | down | 0.011 |
| mmu-miR-290b-3p | 1.990 | down | 0.033 |
| mmu-miR-129b-5p | 1.871 | up | 0.023 |
| mmu-miR-1187 | 1.671 | up | 0.011 |

Note: [H] means HFD-Exos; [N] means NCD-Exos.

**Supplementary Table S2. The resource of key reagents and commercial assays.**

| **REAGENT or RESOURCE** | **SOURCE** | **IDENTIFIER** |
| --- | --- | --- |
| **Antibodies** | | |
| Anti-CD3 | BioLegend | Cat# 100243 |
| Anti-CD9 | BioLegend | Cat# 124802 |
| Anti-CD49b | BioLegend | Cat# 103501 |
| Anti-HSP70 | Lianke | Cat# RK-200-301-A27 |
| Anti-CD63 | Wanleibio | Cat# [WL02549](http://shop.wanleibio.cn/Product/ProductIntroduce?pid=624) |
| Anti-GRP94 | Wanleibio | Cat# [WL04446](http://shop.wanleibio.cn/Product/ProductIntroduce?pid=1969) |
| Anti-TSG101 | Wanleibio | Cat# [WL05130](http://shop.wanleibio.cn/Product/ProductIntroduce?pid=2225) |
| Anti-AKT | Wanleibio | Cat# [WL0003b](http://shop.wanleibio.cn/Product/ProductIntroduce?pid=236) |
| Anti-pAKT | Wanleibio | Cat# [WLP001a](http://shop.wanleibio.cn/Product/ProductIntroduce?pid=889) |
| Anti-PPARγ | Wanleibio | Cat# [WL01800](http://shop.wanleibio.cn/Product/ProductIntroduce?pid=432) |
| Anti-GLUT4 | Wanleibio | Cat# [WL02425](http://shop.wanleibio.cn/Product/ProductIntroduce?pid=780) |
| Anti-NK-κB p65 | Wanleibio | Cat# [WL01273b](http://shop.wanleibio.cn/Product/ProductIntroduce?pid=412) |
| Anti-p-NK-κB p65 | Wanleibio | Cat# [WL02169](http://shop.wanleibio.cn/Product/ProductIntroduce?pid=827) |
| Anti-SMAD6 | abcam | Cat# ab273106 |
| **Chemicals, Peptides, and Recombinant Proteins** | | |
| high-glucose DMEM medium | HyClone | Cat#SH30243.01B |
| FBS | Sigma-Aldrich | Cat#12103C |
| dexamethasone | Sangon Biotech | Cat# A601187 |
| 3-isobutyl-1-methylxanthine | Sangon Biotech | Cat# A606630 |
| insulin | Sigma-Aldrich | Cat# I9278 |
| DMEM/F12 medium | Invitrogen | Cat# 11320033 |
| ITS | Sigma-Aldrich | Cat# I3146 |
| RIPA buffer | KeyGEN BioTECH | Cat#KGP702-100 |
| bicinchoninic acid (BCA) assay | KeyGEN BioTECH | Cat# KGPBCA |
| non-fat milk | BBI | Cat#A600669-0250 |
| Tween-20 | Sangon Biotech | Cat# A100777 |
| PVDF membranes | Immobilon-P | Cat#ISEQ00010 |
| BeyoECL Plus | Beyotime | Cat# P0018S |
| 1640 medium | Biological Industries | Cat#01-100-1ACS |
| 0.22 μm filters | Millipore | Cat#MPGL04GH2 |
| L-sodium lactate | Source leaf organism | Cat#T84071 |
| sodium pyruvate | Solarbio Life Science | Cat# SP0100 |
| N-2-hydroxyethylpiperazine-N-ethane-sulphonicacid | Sangon Biotech | Cat# A600650 |
| lipofectamine 3000 | Invitrogen | Cat#L3000015 |
| TRIzol reagent | Invitrogen | Cat#15596026 |
| HiScript III RT SuperMix | Vazyme | Cat#R323-01 |
| ChamQ Universal SYBR qPCR Master Mix | Vazyme | Cat#Q711-02 |
| EvaGreen miRNA qPCR Master Mix | abm | Cat#G891-1 |
| Protein A dynabeads | Invitrogen | Cat#10001D |
| **Critical Commercial Assays** | | |
| PKH26 Red Fluorescent Cell Linker Kits | Sigma-Aldrich | Cat# PKH26PCL |
| MycoAlert Mycoplasma Detection Kit | Lonza | Cat# LT07-318 |
| glycated hemoglobin kit | Nanjing Jiancheng Bioengineering Institute | Cat# A056-1 |
| glycated serum protein detection kit | Nanjing Jiancheng Bioengineering Institute | Cat# A037-1 |
| triglyceride (TG) assay kit | mlbio | Cat# ml076637 |
| glucose detection kit | Nanjing Jiancheng Bioengineering Institute | Cat# F006-1-1 |
| Dual Luciferase kit | Promega | Cat# E2920 |
| miRNA cDNA Synthesis Kit | abm | Cat# G898 |
| IL-1β ELISA Kit | Annuoruikang | Cat# TAE-370m |
| IL-6 ELISA Kit | Annuoruikang | Cat# TAE-385m |
| TNF-α ELISA Kit | Annuoruikang | Cat# TAE-569m |

**Supplementary Table S3. The sequences of miR-1249-3p mimic, miR-NC, miR-1249-3p inhibitor, inh-NC and siRNA.**

|  | Sense (5′-3′) | Antisense (5′-3′) |
| --- | --- | --- |
| miR-1249-3p mimic | ACGCCCUUCCCCCCCUUCUUCA | AAGAAGGGGGGGAAGGGCGUUU |
| miR-NC | UUCUCCGAACGUGUCACGUTT | ACGUGACACGUUCGGAGAATT |
| miR-1249-3p inhibitor | CAGUACUUUUGUGUAGUACAA | / |
| inh-NC | CAGUACUUUUGUGUAGUACAA | / |
| Si-SKOR1 | CGUCAAGGCUAUGUUUAAUTT | AUUAAACAUAGCCUUGACGTT |
| si-SMAD6 | CUACUCAACAACCACAGAUTT | AUCUGUGGUUGUUGAGUAGTT |
| si-NC | UUCUCCGAACGUGUCACGUTT | ACGUGACACGUUCGGAGAATT |

**Supplementary Table S4. The sequences of qPCR primers.**

| Primer Name | Sequence（5′-3′） |
| --- | --- |
| β-actin-F | ACAGCTTCTTTGCAGCTCCT |
| β-actin-R | GCTGGCATCTGGGTCATCTT |
| Skor1-F | TGAAAGGGACGAACACGTGA |
| Skor1-R | GACTCTGGAATTCCCGCTCC |
| SMAD6-F | CTACTCGCGACAGTTCATCAC |
| SMAD6-R | GCAGCCGCATTGCTATCT |
| IL-6-F | TCTCTCTGAAGGACTCTGGCT |
| IL-6-R | TCCAGTTGCCTTCTTGGGAC |
| IL-1β-F | TGGGTGTGCCGTCTTTCATT |
| IL-1β-R | CCAGCTTCAAATCTCGCAGC |
| TNF-α-F | AAGGTACAACCCATCGGCTG |
| TNF-α-R | AGGCACTCCCCCAAAAGATG |
